# Early assessment of the severity of asphyxia in term newborns using parameters of blood count

**DOI:** 10.2478/v10102-010-0043-x

**Published:** 2010-11

**Authors:** Ingrid Brucknerová, Eduard Ujházy, Michal Dubovický, Mojmír Mach

**Affiliations:** 1 1^st^ Pediatric Hospital, School of Medicine, Comenius University, Bratislava, Slovak Republic; 2 Institute of Experimental Pharmacology, Slovak Academy of Sciences, Bratislava, Slovak Republic

**Keywords:** asphyxia, newborns, hemoglobin, erythrocyte, hematocrit, thrombocyte, leukocyte

## Abstract

Acute perinatal asphyxia is a major cause of death and neurological injury in newborn infants. Severe asphyxia can occur in infants around the time of birth for several reasons. The aim of our study was to find the most sensitive, easily obtainable and fast assessable parameter of the presence and quantification of asphyxia.

In our study 39 term newborns (15 healthy term newborns and 24 asphyxial term newborns), from vaginal deliveries admitted within 24 hours of life were monitored and parameters of blood count from venous blood were assessed. Laboratory findings of blood count parameters revealed significant differences between term asphyxial and healthy newborns in erythrocyte count and hemoglobin and hematocrit values.

Hematological changes observed early after delivery can determine the duration of hypoxemia (acute vs. chronic) and asphyxia of short duration may be accompanied without occurrence of polyglobulia.

## Introduction

The fetal tissues, which are adapted to a low oxygenation during intrauterine development, are subjected to a rapid change in an oxygen concentration after delivery. Birth itself is a strong oxidative stress. Oxidative stress, excess of free radicals and their reactive metabolites in the organism, plays an important role in the pathogenesis of so called free-radical diseases, not only in adults but also in newborns (Brucknerová *et al*., 2007; Tsukahara, 2007). In the case of preterm newborns the consequences of asphyxia are even more threatening due to the immaturity of enzymatic and non-enzymatic antioxidant mechanisms. Postasphyxial complications have a broad spectrum (Brucknerová & Benedeková, 2000; Brucknerová *et al*., 2005).

Changes in the hemopoietic system can be observed as complications of asphyxia. Altered biophysical characteristics of blood, e.g. changes in the structure and function of erythrocytes, leukocytes and thrombocytes can be caused be asphyxia (Curtin *et al*., 2002; Bracci *et al*., 2006).

Hematologic adaptations in the fetus include long- and short-term mechanisms (Rolfo *et al*., 2007). Polycythemia is not only a consequence of intrauterine growth retardation, but may be caused also by asphyxia. During chronic hypoxemia, polycythemia is developed as an adaptation to lack of oxygen supply. Due to decreased tissue oxygenation, increased production of erythropoietin by the kidney is followed by an increasing number of erythrocytes and raised fetal blood volume. If hypoxemia is present also during labor, hemoconcentration develops. Polycythemia/hemoconcentration changes blood biophysical characteristics, with elevated values of blood viscosity. Hyperviscosity has a negative influence on the postnatal newborn's adaptation and affects blood distribution between organs. The higher the viscosity, the lower the blood flow in the organism (central nervous system, cardiovascular, respiratory or urinary system), and the higher the risk of thrombosis, mainly in the CNS, increased minute volume and increased right-to-left shunt. On the other hand, acute hypoxemia involves short-term responses like reduction of body temperature, reduction of heart rate and/or redistribution of circulation (Singer, 1999). Hematological changes observed early after delivery can determine the duration of hypoxemia (acute vs. chronic) and help to assess therapeutic strategy.

## Patients and methods

The study involved 39 term newborns (15 healthy term newborns, HTN, birth weight 3675±765 g and 24 asphyxial term newborns, ATN, birth weight 2752±144.5 g), from vaginal deliveries admitted within 24 hours of life. In both groups of newborns, blood samples of venous blood were taken on the 1^st^ and 5^th^ day of life and parameters of blood count (erythrocytes, hemoglobin, hematocrit, leukocytes, and thrombocytes) were determined.

Blood count was determined by an automatic analyzer ADVIA 120 (Bayer Diagnostics, New York, USA) using an optical system for measurement. The number of erythrocytes, leukocytes and thrombocytes was assessed by the analyzer using a laser beam which registered the flow of cells in certain time intervals. Hemoglobin was measured colorimetrically. Using a laser beam, the size of erythrocytes was determined and further the value of hematocrit was calculated.

For statistical analysis ANOVA followed by Fisher's post hoc test was used, *p* ≤ 0.05 was considered significant. The presence of asphyxia was the first factor (asphyxial term newborns, healthy term newborns) and the time of blood sampling was the second factor (1^st^ and 5^th^ day of life).

This study complies with the guidelines for human studies and animal welfare regulations and subjects have given their informed consent. The study protocol has been approved by the Committee on Human Research in Medical School, Comenius University, Bratislava, Slovakia.

## Results

Laboratory findings of blood count parameters revealed significant differences between term asphyxial and healthy newborns in erythrocyte count and hemoglobin and hematocrit values (Table 1). We did not observe any differences between these groups in leukocyte and thrombocyte counts.

**Table 1:** Blood count results.

Parameter	1^st^ day of life		5^th^ day of life	
		
	Term healthy newborns	Term asphyxial newborns	Term healthy newborns	Term asphyxial newborns
	(n=15)	(n=24)	(n=15)	(n=24)
Leukocytes	19.61±1.44	18.83±1.66	11.82±1.57	10.27±1.10
Erythrocytes	5.22±0.14	4.38±0.12^**^	5.03±0.13	4.14±0.12^***^
Hemoglobin	187.13±5.88	160.66±5.4^**^	178.80±5.28	147.09±5.03^***^
Hematocrit	0.53±0.01	0.45±0.01^**^	0.50±0.01	0.41±0.01^***^
Thrombocytes	255.26±15.07	231.25±17.59	274.06±14.73	239.95±21.68

** *p* ≤ 0.01;

*** *p* ≤ 0.001-statistically significant difference between both groups on the same day of life

## Discussion

Several changes in structure, function and amount of certain blood elements have been observed in asphyxia (Curtin *et al*., 2002; Bracci *et al*., 2006).

In the asphyxia, the increased production of erythropoietin increases the amount of nucleated erythrocytes, from which the non-nucleated cells matured gradually. The presence of nucleated erythrocytes in umbilical blood confirms the presence of an asphyxial event during intrauterine development (Curtin *et al*., 2002; Bracci *et al*., 2006). An increased amount of nucleated erythrocytes is the consequence of chronic intrauterine asphyxia (Phelan *et al*., 1995; Fahnenstich *et al*., 1995).

However in acute asphyxia, another physiological adaptation mechanism is present. This mechanism, activated by asphyxia, consists in shunting blood from the skin and splanchnic area to the heart, adrenals, and brain. This recirculation of blood ostensibly protects these vital organs from hypoxic-ischemic injury.

In our study blood count variables were found to be affected differently than observed by other authors. In the blood samples obtained on the 1^st^ day of life, polyglobulia as a sign of chronic fetal hypoxia failed to be confirmed not even in a single case. After evaluation of red blood components in peripheral blood on the 1^st^ day of life, we found a statistically significant decrease of erythrocytes and hemoglobin in asphyxial term newborns. Anemia required transfusion of erythrocytes in 4 newborns. We presumed that anemia was caused by intensification of oxidative stress during prenatal and direct postnatal period, as well as by blood redistribution or by hemorrhage. In 20 asphyxial term newborns we assumed the participation of adaptive mechanisms which help maintain optimal blood supply to the most sensitive organs (CNS, heart).

In asphyxia, in the first 96h of life increased peripheral leukocyte counts may contribute to abnormal neurodevelopmental outcome (Morkos *et al*., 2007). In our study we did not find any differences in leukocyte counts between HTN and ATN. During the first week of life the number of leukocytes decreased in both groups, indicating physiological changes normally seen in healthy term newborns. We can only assume that the asphyxia in our group of patients was of too short duration and low severity to produce changes observed by Morkos *et al*. (2007).

Thrombocytes take part in hemostasis, influence coagulation cascade and are the main source of many biologically active substances. Thrombocytopenia can be observed during the first 2 days of life, on the 10^th^ day of life a normal number of thrombocytes is established.

In either groups studied we did not confirm a statistically significant thrombocytopenia, though in the group of asphyxial newborns the levels of thrombocytes were slightly decreased on 1^st^ and 5^th^ day of life. We agree with the finding of Phelan *et al*. (2007), that thrombocytopenia is inconsistent with acute birth asphyxia and therefore is not a sensitive marker in the case of a mild degree of acute asphyxia.

## Conclusion

In spite of the recent decrease of perinatal morbidity and mortality, the risk of pre/postnatal CNS damage as a consequence of asphyxia did not decrease. The aim of current perinatology research is finding and determining the most sensitive, easily obtainable and fast assessable parameter of the presence and quantification of asphyxia.

The newborns have inadequate antioxidant protection and oxidative stress is a major cause of irreversible CNS damage in term asphyxial newborns. Studies have confirmed the helpful effects of antioxidant supplementation during hypoxemia (Pecháň *et al*., 1996; Holomáň & Pecháň, 2002; Holomáň *et al*., 1999; Vento *et al*., 2003; Shoji & Koletzko, 2007). Our results showed the presence of asphyxia of short duration to be without occurrence of polyglobulia as a sign of chronic hypoxia, and that by using simple blood tests commonly applied by neonatologists. Hematological changes observed early after delivery can determine the duration of hypoxemia (acute vs. chronic). These laboratory findings are often vital for neonatologist in case of unknown history of fetal asphyxia.

In combination with other biochemical blood parameters (Brucknerová *et al*., 2007; Farkouh *et al*., 2006) and clinical signs we may predict the severity of the asphyxial insult and decide on the relevant therapeutic strategy and management of newborns.
